# Enhanced cardiac *TBC1D10C* expression lowers heart rate and enhances exercise capacity and survival

**DOI:** 10.1038/srep33853

**Published:** 2016-09-26

**Authors:** Cornelia Volland, Sebastian Bremer, Kristian Hellenkamp, Nico Hartmann, Nataliya Dybkova, Sara Khadjeh, Anna Kutschenko, David Liebetanz, Stefan Wagner, Bernhard Unsöld, Michael Didié, Karl Toischer, Samuel Sossalla, Gerd Hasenfuß, Tim Seidler

**Affiliations:** 1 Department of Cardiology and Pulmonology, Georg-August-University, Robert-Koch Str. 40, 37075 Göttingen, Germany; 2 Department of Clinical Neurophysiology, Georg-August-University, Robert-Koch Str. 40, 37075 Göttingen, Germany; 3 Department of Internal Medicine II, University Hospital Regensburg, Franz-Josef-Strauß-Allee 11, 93053 Regensburg, Germany; 4 Institute of Pharmacology, Georg-August-University, Robert-Koch Str. 40, 37075 Göttingen, Germany

## Abstract

TBC1D10C is a protein previously demonstrated to bind and inhibit Ras and Calcineurin. In cardiomyocytes, also CaMKII is inhibited and all three targeted enzymes are known to promote maladaptive cardiomyocyte hypertrophy. Here, in accordance with lack of Calcineurin inhibition *in vivo*, we did not observe a relevant anti-hypertrophic effect despite inhibition of Ras and CaMKII. However, cardiomyocyte-specific TBC1D10C overexpressing transgenic mice exhibited enhanced longevity. Ejection fraction and exercise capacity were enhanced in transgenic mice, but shortening of isolated cardiomyocytes was not increased. This suggests longevity resulted from enhanced cardiac performance but independent of cardiomyocyte contractile force. In further search for mechanisms, a transcriptome-wide analysis revealed expressional changes in several genes pertinent to control of heart rate (HR) including *Hcn4*, *Scn10a*, *Sema3a and Cacna2d2.* Indeed, telemetric holter recordings demonstrated slower atrial conduction and significantly lower HR. Pharmacological reduction of HR was previously demonstrated to enhance survival in mice. Thus, in addition to inhibition of stress signaling, TBC1D10C economizes generation of cardiac output via HR reduction, enhancing exercise capacity and survival. TBC1D10C may be a new target for HR reduction and longevity.

A large body of work exists aimed at inhibition of maladaptive cardiac signalling to rescue heart failure and pathological remodeling. The Ras/MAPK- and the Calcineurin (CaN)/NFAT signalling pathways are the most intensively studied and are undoubtedly involved in cardiac pro-hypertrophic signalling. Although experimental data have been promising, no suitable therapeutic concept could be developed for human heart failure or hypertrophy based on direct inhibition of CaN or Ras/MEK/ERK. However, targeting multiple pathways in parallel may open up new opportunities.

Here, we investigated the effects of transgenic overexpression of TBC1D10C (also known as Carabin and EPI64C) in mouse myocardium. The primary structure of TBC1D10C contains a Ras GTPase activating protein (GAP) domain at its N-terminus and a CaN interacting domain^1^,^2^. Accordingly, it has been demonstrated that TBC1D10C physically interacts with H-Ras and CaN in T cells, inhibits Ras/MAPK signalling and, moreover, is a negative feedback inhibitor of the CaN signalling pathway^1^. As both the CaN and the Ras/MAPK pathways are significantly involved in cardiac hypertrophic signalling^3^,^4^,^5^, our initial aim was to examine the antihypertrophic potential of enhanced TBC1D10C expression in cardiomyocyte specific transgenic (TG) mice. In our model a significant antihypertrophic effect could be excluded. Instead, a unique phenotype with different beneficial cardiac effects, e.g. higher ejection fraction, exercise capacity and longevity was associated with cardiomyocyte specific TG TBC1D10C expression.

## Results

### Hearts of *TBC1D10C* TG mice display increased fractional shortening and reduced heart rate

**Figure 1:**
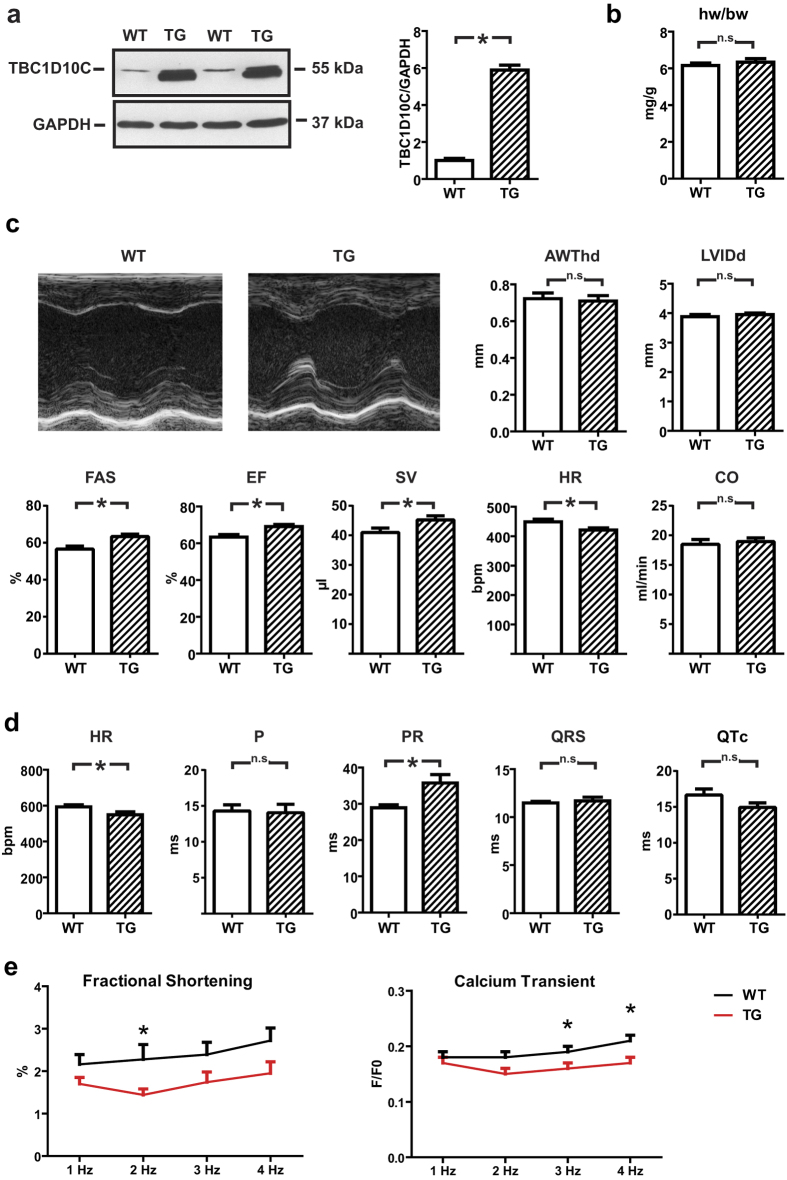
Phenotype of *TBC1D10C* TG mice. (**a**) Western blot displaying overexpression levels of TBC1D10C in WT vs. TG mice in myocardial tissue (*n* = 4). The full length membrane of this cropped blot is displayed in Supplemental Fig. 6. (**b**) Heart weight to body weight ratio (hw/bw) (WT: *n* = 16, TG: *n* = 12; n.s.). (**c**) Echo-cardiography in 9-week-old mice revealed similar anterior wall thickness (AWThd) and left ventricular inner diameter (LVIDd), significantly increased fractional area shortening (FAS), ejection fraction (EF), stroke volume (SV), reduced heart rate (HR) and similar cardiac output (CO) in the TG (WT: *n* = 33, TG: *n* = 39 mice; *P* < 0.05). (**d**) Telemetric ECG recordings in conscious mice confirmed significantly reduced heart rate (HR) and revealed a prolonged PR interval and equal QRS and QTc intervals in *TBC1D10C* TG vs. WT mice (*n* = 7 per group; *P* < 0.05). (**e**) Fractional shortening (left) and calcium transient measurements in isolated left ventricular cardiomyocytes from WT and TG mice (*n* = 7 mice per group, 6–10 cells each; *P* < 0.05; two-way ANOVA).

We generated TG mice with moderately enhanced cardiomyocyte-specific expression of TBC1D10C (Fig. 1a). TG mice were healthy and of equal weight and their hearts did not exhibit macroscopic differences. Echocardiographic analysis of a large cohort of 9-week-old mice revealed a substantial increase in fractional area shortening (FAS) (wild-type [WT]: 56.6 ± 1.6%, *n* = 33; TG: 63.4 ± 2.0%, *n* = 39; *P* < 0.005), ejection fraction (EF) (WT: 63.3 ± 1.4%, *n* = 33; TG: 69.1 ± 1.1%, *n* = 39; *P* < 0.005) and stroke volume (SV) (WT: 41.0 ± 1.5 μL, *n* = 33; TG: 45.2 ± 1.4 μL, *n* = 39; *P* < 0.05) whereas heart rate (HR) was significantly decreased (WT: 449 ± 9 beats/min, *n* = 33; TG: 421 ± 7 beats/min, *n* = 39; *P* < 0.05) (Fig. 1b,c). The reciprocal relationship between HR and SV resulted in a neutral effect on echocardiographically determined cardiac output (CO) (WT: 18.5 ± 0.8 mL/min, *n* = 33; TG: 19.0 ± 0.6 mL/min, *n* = 39; n.s.). Of note, structural and morphological parameters (left ventricular internal diameter at end diastole [LVIDd], diastolic anterior wall thickness [AWThd] and heart weight/body weight ratio [HW/BW]) remained identical between WT and TG littermates. This phenotype was largely preserved in a second, independently generated TG mouse line with relatively lower expression of TBC1D10C (Supplemental Fig. 1a,b), in which FAS and EF were also significantly enhanced. HR was also reduced in this second line (line 2); although the reduction did not reach statistical significance (HR: WT: 407 ± 12 beats/min, *n* = 15; TG: 384 ± 13 beats/min, *n* = 15; n.s.), a similar CO was observed in WT and TG mice despite significantly higher EF (WT: 54.1 ± 2.3%, *n* = 15; TG: 60.7 ± 2.8%, *n* = 15; *P* < 0.05) (Supplemental Fig. 1c). Owing to lower TBC1D10C overexpression, less reduction of HR, and less increase in SV in line2, line1 was chosen for additional studies.

In view of the lower HR during echocardiography in TG mice, we sought to exclude effects of anaesthesia and to evaluate further the role of TBC1D10C in cardiac conduction. For this purpose, electrocardiography (ECG) data were acquired from conscious WT and *TBC1D10C* TG littermates with subcutaneously implanted radiotelemetry transmitters. These experiments confirmed a significant reduction in ambulatory HR compared to WT littermates (WT: 592 ± 11 beats/min; TG: 549 ± 16 beats/min; *P* < 0.05; *n* = 7 per group). In addition, TG mice exhibited significant prolongation of the PR interval (WT: 28.9 ± 0.8 ms; TG: 35.8 ± 2.3 ms; *P* < 0.05; *n* = 7 per group) whereas P-wave, QRS and QTc durations were similar to WT mice (Fig. 1d). To examine whether altered regulation of the autonomous nervous system might be involved in the heart rate reduction of transgenic animals, we measured heart rate variability from the telemetric recordings. Time-domain analysis showed a significant reduction in the heart rate variability (Suppl. Table 1). Interestingly, frequency-domain analysis revealed unchanged activity of the parasympathetic nervous system, while sympathetic regulation of transgenic hearts was disturbed. The normalized low frequency (LF) was significantly reduced (WT: 30.4 ± 4.1 nu; TG: 18.0 ± 3.8 nu; P < 0.05; n = 6 per group) but normalized high frequency (HF) was unaltered (WT: 69.3 ± 4.0 nu; TG: 70.5 ± 7.5 nu; P = N.S.; n = 6 per group). As a consequence, frequency domain ratio (LF/HF) was significantly reduced (WT: 0.49 ± 0.07; TG: 0.28 ± 0.06; P < 0.05; n = 6 per group) (Supplemental Table 1). Isolated cardiomyocytes from TG mice revealed marginally reduced shortening and Ca^2+^ transients. This important *in vitro* finding strongly suggests that enhanced FS, EF, and SV observed *in vivo* are not a primary effect of cardiomyocytes but compensatory reaction towards lower heart rate (Fig. 1e).

### *TBC1D10C* TG mice are not protected from TAC- or AngII-induced hypertrophy

**Figure 2:**
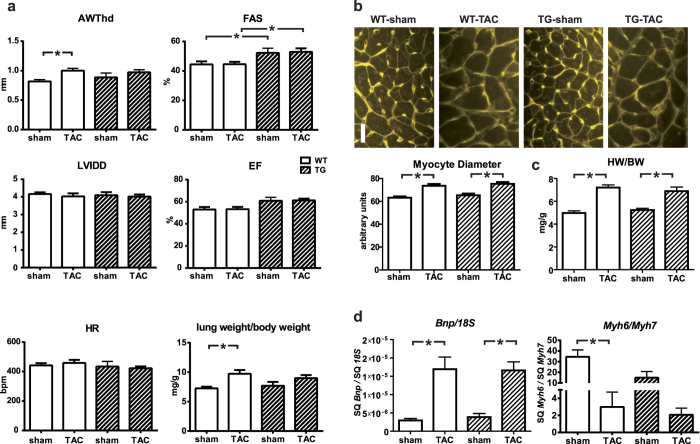
Response of *TBC1D10C* TG mice towards aortic banding (TAC)–induced hypertrophy. (**a**) 8-week-old *TBC1D10C* TG mice underwent TAC intervention with a 27G needle or were sham operated. Echocardiography 8 weeks after surgery revealed significantly increased diastolic anterior wall thickness (AWThd) in WT TAC vs. WT sham mice, but there was no difference between WT and TG, or TG sham vs. TG TAC animals. Left ventricular inner diameter (LVIDd) was not different, and fractional area shortening (FAS) and ejection fraction (EF) were increased in TG vs. WT. (*n* = 9–17 mice per group; *P* < 0.05). Morphometry 8 weeks after the TAC intervention demonstrates increased lung weight as a sign of pulmonary congestion secondary to heart failure in TAC vs. sham operated animals. (Note: Echocardiography at 4 weeks after TAC is shown in supplemental Fig. 3). (**b**) WGA-stained myocardial sections were used for measurement of cardiomyocyte diameter. WT and TG mice displayed equally induced cellular hypertrophy. Scale bar: 20 μm (*n* = 7–9). (**c**) Heart weight/body weight was significantly increased after 8 weeks of TAC. (*n* = 7–15). (**d**) Real-time RT-PCR revealed a significant increase in *Bnp* expression and a decrease of *Myh6*/*Myh7* after 8 weeks of TAC, but no significant differences between WT and TG (*n* = 7–13). (SQ: template starting quantity.)

**Figure 3:**
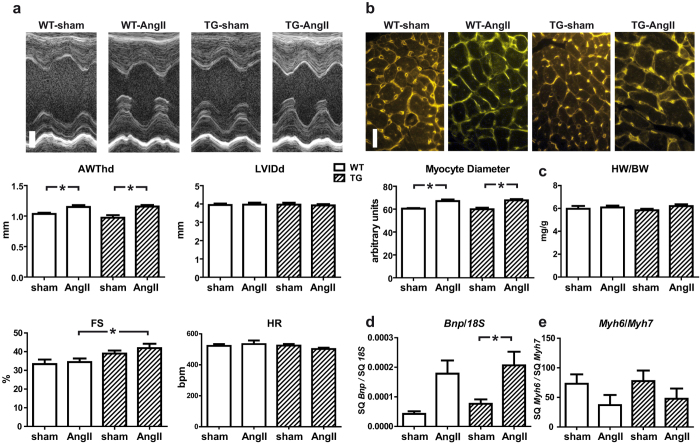
Response of *TBC1D10C* TG mice towards angiotensin II-induced hypertrophy. (**a**) WT and *TBC1D10C* TG mice underwent chronic angiotensin II (AngII) infusion using micro-osmotic pumps. Original echocardiographic recordings after 2 weeks of AngII treatment (top; scale bar: 1 mm) and cumulative data (below). Diastolic anterior wall thickness (AWThd) was significantly increased after AngII treatment, whereas left ventricular inner diameter (LVIDd) was unchanged. Fractional shortening (FS) was increased in TG mice after AngII treatment (WT sham: *n* = 6; WT AngII: *n* = 9; TG sham: *n* = 6; TG AngII: *n* = 9). (**b**) Wheat germ agglutinin (WGA) stain of cardiac sections revealed a significant increase in cardiomyocyte diameter in AngII-treated mice, but no differences between WT and TG (*n* = 6–8 mice, 100 cells each, *P* < 0.05, one-way ANOVA). Scale bar: 20 μm. (**c**) Heart weight to body weight ratio was not increased after 2 weeks of AngII treatment (WT sham: n = 6; WT AngII: *n* = 9; TG sham: *n* = 6; TG AngII: *n* = 9). (**d**) Quantitative real-time RT-PCR of *Bnp* mRNA expression normalized to 18S RNA and the ratio of α- (*Myh6*) to β- (*Myh7*) myosin heavy chain gene transcript levels (WT sham: *n* = 6; WT AngII: *n* = 9; TG sham: *n* = 6; TG AngII: *n* = 9; *P* < 0.05; one-way ANOVA). (SQ: template starting quantity.)

In isolated cardiomyocytes with adenoviral mediated overexpression of *TBC1D10C* we observed significantly impeded CaN signalling and diminished hypertrophic growth in response to phenylephrine (PE) and angiotensin II (AngII) treatment (Supplemental Fig. 2). We hypothesized that *TBC1D10C* TG mice would display a similarly decreased hypertrophic response. Accordingly, *TBC1D10C* TG mice and WT littermates underwent aortic banding surgery. Four weeks after transverse aortic constriction (TAC) anterior wall thickness (AWThd) was significantly increased (WT sham: 0.69 ± 0.03 mm, *n* = 20; WT TAC: 0.93 ± 0.04 mm, *n* = 23; *P* < 0.05), while echocardiography displayed no signs of dilatation or functional impairment (Supplemental Fig. 3). However, the hypertrophic response towards increased afterload by TAC was similar in *TBC1D10C* TG mice compared to WT at 4 weeks (WT TAC: 0.93 ± 0.04 mm, *n* = 23; TG TAC: 0.96 ± 0.04 mm, *n* = 19; n.s.) and at 8 weeks (Fig. 2), although hypertrophy, increase in lung weight and percentage of mice with lung edema were somewhat less pronounced in TG compared to WT mice subjected to TAC (assuming a threshold of 10 mg/g, 8 of 19 WT animals (42%) and 5 of 17 TG animals (29%) exhibited lung edema 8 weeks after TAC). Furthermore, both groups had a similar increase in myocyte diameters measured via wheat germ agglutinin (WGA) staining and similar heart weight/body weight ratios at 8 weeks after TAC (Fig. 2b,c) excluding marked protection from maladaptive hypertrophy in this model. Likewise, the expression of both the B-type natriuretic peptide gene (*Bnp*) and of *Myh6/Myh7* was induced equally in both groups after TAC (Fig. 2d), clearly demonstrating that these *TBC1D10C* TG mice are not protected from TAC-induced hypertrophy or heart failure. Moreover, to exclude the possibility of a blunted antihypertrophic effect due to higher transaortic gradients with enhanced SV in TG mice, we evaluated whether TBC1D10C was antihypertrophic in mice subjected to chronic AngII infusion utilizing implanted micro-osmotic pumps. Consistent with the observations in the TAC model, *TBC1D10C* TG mice did not display a blunted hypertrophic response after AngII treatment, as demonstrated by echocardiography, WGA staining, and real-time reverse transcriptase-polymerase chain reaction (RT-PCR) of *Bnp* and *Myh6/Myh7* expression (Fig. 3). To evaluate the effect of TBC1D10C in a model of more severe heart failure compared to TAC we induced myocardial infarction (MI) by permanent ligation of the left anterior descending artery (LAD). Mice exhibited large infarctions and clinical signs of heart failure. At two weeks after MI surgery, mice exhibited large akinetic areas with thinning of the myocardium extending towards the inferior wall and severely impaired contractile function (Supplemental Fig. 4a,b). Although heart weight and echocardiography did not reveal marked differences in WT compared to TG mice, myocyte cross sectional (transversal) diameters determined with WGA staining in unaffected regions of the LV revealed a small difference between the genotypes, suggesting a modest anti-hypertrophic effect of TBC1D10C in this model. Likewise, in a healthy cohort of mice 12 month of age there was a small statistically significant difference in cross sectional myocyte diameter in WGA stain although this did not translate to significant differences in echocardiographic or morphometric parameters. Staining for fibrosis was similar in aged mice (Supplemental Fig. 4c,d).

### Ras activity and CaMKII phosphorylation are diminished in *TBC1D10C* TG mice

**Figure 4:**
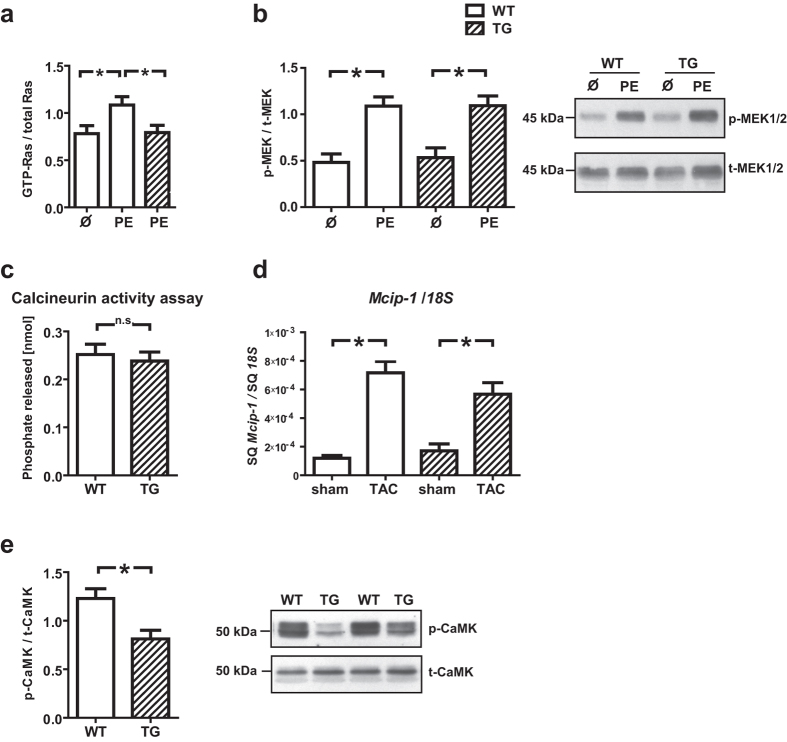
Examination of Ras/MAPK, CaMKII and CaN activity. (**a**) Ras activity was significantly reduced in *TBC1D10C* TG vs. WT mice (after phenylephrine (PE) stimulation) (WT, uninjected: *n* = 4; WT + PE: *n* = 14; TG + PE: *n* = 15; *P* < 0.05; Student’s *t*-test), whereas (**b**) p-MEK1/2/t-MEK1/2 levels were not significantly diminished in PE stimulated TG vs. PE stimulated WT mice (WT, uninjected: *n* = 3; WT + PE: *n* = 15; TG, uninjected: *n* = 3; TG + PE: *n* = 15,n.s.; Student’s *t*-test). (**c**) Calcineurin (CaN) phosphatase activity was not different between WT and TG mice (*n* = 6 per group), suggesting CaN-independent signal pathways may be more important for the effects of TBC1D10C *in vivo*. (**d**) *Mcip-1* (Modulatory calcineurin-interacting protein 1) mRNA expression, a marker for CaN activity, was not significantly changed in TG mice whereas (**e**) CaMKII (T286) phosphorylation was significantly diminished in TG mice (TG: *n* = 8; WT: *n* = 10; *P* < 0.01; Student’s *t*-test). Full length blots of the cropped membranes in Fig. 4b and e are displayed in Supplementary Figure 6.

In view of our *in vitro* data and previously published work demonstrating that TBC1D10C overexpression inhibits Ras/MAPK, CaMKII, and CaN, we addressed the lack of an physiologically significant antihypertrophic effect in our models by validating these signalling pathways also *in vivo*. We observed decreased Ras activity in *TBC1D10C* TG mice compared to WT littermates after 15 min of stimulation with phenylephrine (GTP-Ras/total Ras: WT uninjected: 0.78 ± 0.08, *n* = 4; WT+PE: 1.08 ± 0.09, *n* = 14; TG+PE: 0.79 ± 0.08, *n* = 15; *P* < 0.05). However, diminished Ras activity did not provoke a significant change in MEK1/2 phosphorylation (Fig. 4a,b). Furthermore, *TBC1D10C* TG mice did not exhibit decreased CaN activity (Fig. 4c) or altered *Mcip-1* mRNA expression (no intervention, with TAC or with myocardial infarction) as a marker for CaN activity (Fig. 4d). Instead, CaMKII (T286) phosphorylation was significantly reduced in *TBC1D10C* TG mice (WT: 1.2 ± 0.1, *n* = 8; TG: 0.81 ± 0.09, *n* = 10; *P* < 0.01) (Fig. 4e). Thus, in our TG mice, we did not identify significant inhibition of either CaN or MEK1/2, suggesting this explains the lack of protection against hypertrophy. Notably, inhibition of both Ras and CaMKII activities appear as robust findings on enhanced TBC1D10C expression regardless of the method of overexpression.

### Increased longevity and exercise endurance in *TBC1D10C* TG mice

**Figure 5:**
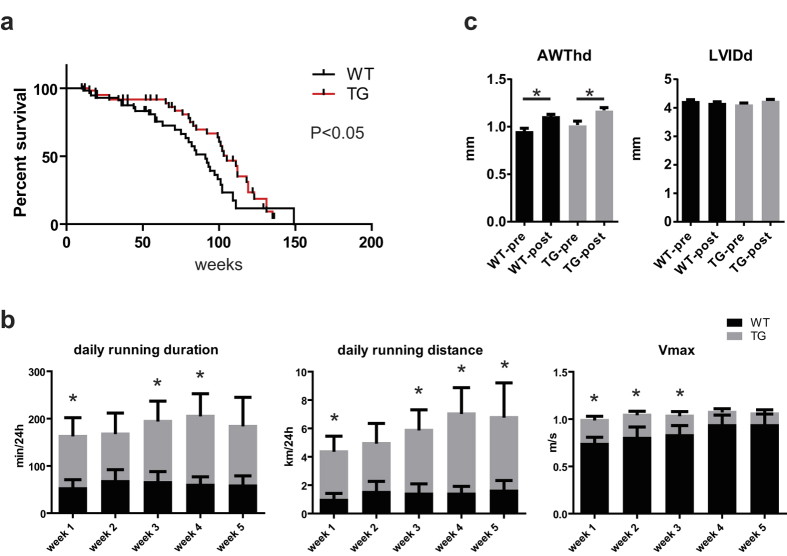
Longevity and exercise endurance in *TBC1D10C* TG mice. (**a**) Kaplan–Meier analysis of survival was determined in a prospectively defined cohort of mice. Spontaneous deaths were recorded (WT: *n* = 79; TG: *n* = 86; *P* < 0.05; log-rank (Mantel–Cox) test). (**b**) Voluntary running was recorded over 5 weeks. TG mice exhibited significantly longer daily running duration at weeks 1, 3 and 4, longer running distance at weeks 1, 3, 4 and 5, and *V*_max_ was significantly increased at weeks 1, 2 and 3 (WT: *n* = 6; TG: *n* = 8; *P* < 0.05). (**c**) Exercise-induced hypertrophy of the anterior wall (anterior wall thickness in diastole (AWthd)) was similar between WT and TG mice. Left ventricular enddiastolic diameter (LVIDD) remained unchanged.

Selective targeting of heart rate by the If channel inhibitor ivabradine was recently demonstrated to enhance longevity in healthy mice. Here, we constantly observed reduced HR that was repeatedly determined until at least 12 months of age (Supplemental Fig. 4e). Therefore we hypothesized that survival might be enhanced in TG mice. Kaplan–Meier analysis of survival was conducted in a prospectively defined cohort of mice. All-cause mortality, including spontaneous death and euthanasia of mice because of sickness, was recorded (Fig. 5a). Indeed, median survival of the TG mice was significantly increased by 14 weeks compared to WT (WT: 91 weeks vs. TG: 105 weeks, *P* < 0.05). As survival was greater at a relatively young age, we asked whether higher exercise capacity in *TBC1D10C TG* mice might explain such robust difference. To address this issue, a separate cohort of mice were housed individually in cages equipped with a running wheel and we recorded voluntary running over 5 weeks. Remarkably, these examinations revealed a significantly longer running distance and duration of *TBC1D10C* TG compared to WT mice (Fig. 5b). Exercise-induced hypertrophy was not different between the genotypes (Fig. 5c).

### Transcriptome analysis in *TBC1D10C* TG mice

**Figure 6:**
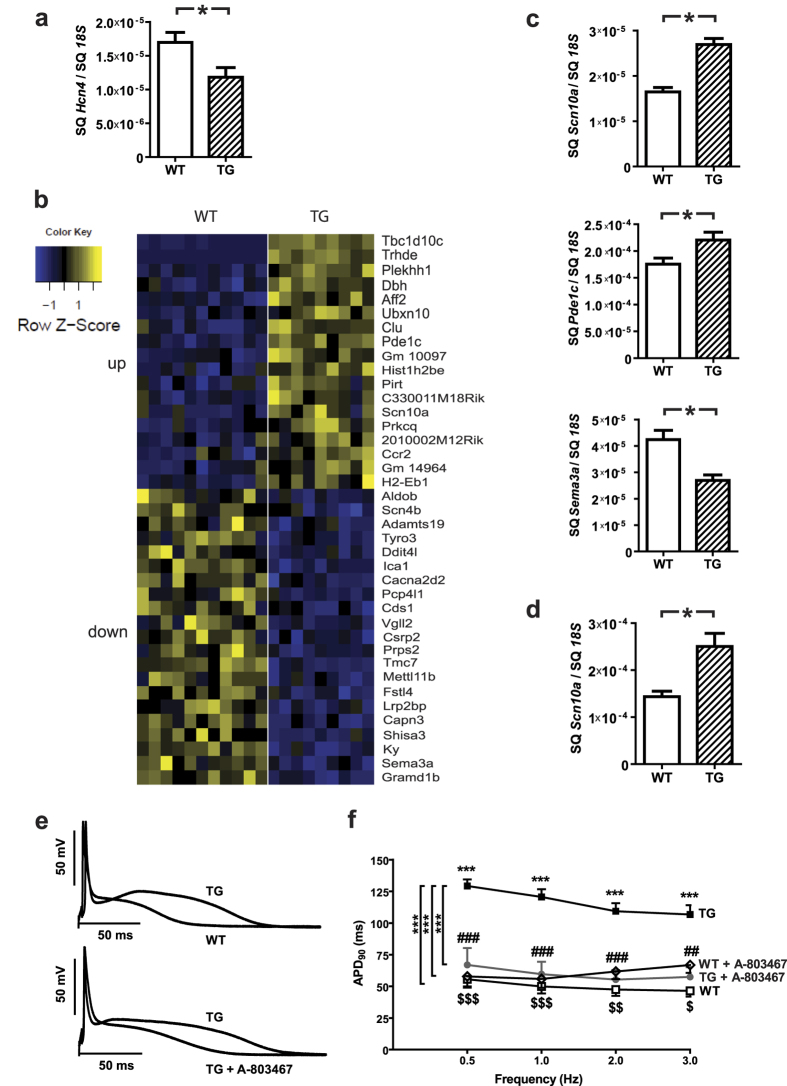
Transcriptome analysis in *TBC1D10C* TG mice. (**a**) *Hcn4* expression levels were significantly reduced in TG vs. WT (*n* = 16 per group; *P* < 0.05). (**b**) Heat map of whole mRNA transcriptome analysis of *TBC1D10C* TG vs. WT mice at the age of 20 weeks. (WT: *n* = 11, TG: *n* = 9; log2-fold change >0.25, *P*(adj) < 0.05). (SQ: template starting quantity.) (**c**) Examples of differentially expressed genes (*Scn10a*, *Pdc1c*, *Sema3a*) validated by quantitative real-time RT-PCR (WT: *n* = 11, TG: *n* = 9). (**d**) Scn10a expression was examined also in atrial myocardium. (**e**,**f**) *TBC1D10C* TG mice exhibited significantly prolonged action potential duration (APD), which was completely reversed by treatment with the specific Scn10a (Na_v_1.8) inhibitor A-803467 (TG: *n* = 9 mice (27 cells); WT: *n* = 6 mice (21 cells); TG + A-803467: *n* = 4 mice (6 cells); WT + A-803467: *n* = 3 mice (6 cells); ***P < 0.0001; two-way RM-ANOVA and Bonferroni-post-test).

We identified significant inhibition of CaMKII and Ras activity in TG mice. However, the sustained beneficial effect of TBC1D10C expression *in vivo* is suggestive of concomitant differences in gene expression. As HCN4 is central to HR regulation, we measured expression of *Hcn4* in TG vs. WT mice. Indeed, *Hcn4* expression was significantly reduced in TG mice (*Hcn4*/*18S*: WT: 1.7 ± 0.1 E-5, *n* = 16; TG: 1.2 ± 0.2 E-5, *n* = 16; *P* < 0.05) (Fig. 6a). To address expression changes in more depth, we then utilized next-generation sequencing (NGS) for whole mRNA transcriptome analysis. Applying a threshold of a log2-fold change >0.25 and the false discovery rate method for multiple testing with a threshold of 5% resulted in 18 upregulated and 21 downregulated genes (Fig. 6b). Among the upregulated genes was *Scn10a (Scn10a*/*18S*: WT: 1.6 ± 0.1 E-5, *n* = 9; TG: 2.7 ± 0.1 E-5, *n* = 10; *P* < 0.0001), coding for the voltage-gated sodium channel Na_V_1.8. *SCN10A* was implicated in HR regulation and, by experimental and genome-wide association studies (GWAS), identified as a modulator of PR interval duration, i.e. atrial conduction time. Moreover, *Sema3a*, also demonstrated to control cardiac conduction and heart rhythm regulation, was significantly downregulated (*Sema3a*/*18S*: WT: 4.2 ± 0.3 E-5, *n* = 10; TG: 2.7 ± 0.2 E-5, *n* = 10; *P* < 0.005). Finally, *Cacna2d2*, also implicated in HR control, was significantly downregulated; however, absolute expression level was extremely low. Differential expression of exemplary genes was confirmed via real-time RT-PCR (Fig. 6c,d). In order to examine patterns of transcriptional regulation which might result in increased lifespan and voluntary exercise capacity of Carabin TG mice, a pathway enrichment analysis was conducted using significantly regulated genes (cut off adjusted p-value < 0.05). The Analysis resulted in a gene ontology/pathway term network showing significant overlapping functional groups (p-value < 0.05) of a number of regulated genes (Supplemental Fig. 5). The analysis suggests that cardiac conduction, regulation of heart rate, regulation of sodium ion transmembrane transport and cardiac muscle contraction are central to the phenotype and closely interconnected. In addition to Scn10a, CaMKII2b and Sema3a, also transcriptional changes of genes encoding Slow skeletal muscle troponin T (Tnnt1), a phospholipid-transporting ATPase (Atp8a2), Calpain 3 (Capn3), the Sodium channel type I beta (Scn1b) and -4 beta (Scn4b), the Na^+^/Ca^2+^ exchanger (Slc8a1 also known as Ncx1), and a number of genes grouping to regulation of sodium ion transmembrane transport (Lrrc15, Vamp5, Pacs1, F11r, Fhl1, Tesc) contributed to the network. However, while this analysis confirms the central role of regulation of cardiac conduction and heart rate for the phenotype of TBC1D10C transgenic mice, the detailed relationships and mechanism of these network components need to be examined in detail in the future.

### *TBC1D10C* TG cardiomyocytes display action potential prolongation via *Scn10a* upregulation

A markedly prolonged PR interval is not a typical feature of reduced HR and was an unexpected finding in telemetrically recorded electrocardiography (ECG) from TG mice and suggests electrophysiological changes induced by enhanced TBC1D10C expression are not limited to sinus node or HCN channels. SCN10A is known to affect both PR interval as well as action potential duration (APD). In view of our data on altered SCN10A expression, to investigate the potential involvement of SCN10A in APD prolongation we measured APD in isolated atrial cardiomyocytes from WT and TG mice. APD90 was significantly prolonged in *TBC1D10C* TG cardiomyocytes at stimulation frequencies from 0.5 to 3 Hz, with the strongest increase at 0.5 Hz (Fig. 6e,f). We measured APD90 in isolated atrial cardiomyocytes in the presence of the specific SCN10A inhibitor A-803467. Our data revealed a marked shortening of APD90 in A-803467–treated *TBC1D10C* TG cardiomyocytes. We observed no significant difference in APD90 between TG and WT cardiomyocytes in the presence of A-803467, demonstrating a substantial contribution of SCN10A to the electrophysiological phenotype (Fig. 6e,f).

## Discussion

Our original findings established that enhanced expression of TBC1D10C *in vivo* evokes (1) a significantly reduced HR associated with parallel regulation of genes regulating sinoatrial node (SAN) function; (2) an increase in fractional shortening and ejection, sufficient to maintain CO despite lower HR; (3) a prolonged APD and PR interval that is associated with enhanced expression and activity of SCN10A in atrial myocytes; (4) enhanced exercise capacity; and (5) enhanced longevity. A second TG line was used to verify the data. Line 2 exhibited reduced TBC1D10C overexpression compared to line 1. Consistent with less enhanced TBC1D10C expression, differences vs. WT were somewhat less pronounced in this line, but HR, EF, FS, and expressional changes exhibited identical trends.

The inverse relationship between HR and EF raises the question of a cause-and-effect relationship. As contractility of isolated cardiomyocytes from TG mice was not enhanced, higher EF is most likely secondary to HR reduction. We observed several distinct alterations in protein expression or activity likely to impact HR in parallel: (1) *Hcn4* mRNA was significantly downregulated in TG mice, suggesting TBC1D10C controls HR via regulation of I_f_; (2) CaMKII activity is reduced. Like I_f_, which is central to regulation of HR at the level of the sarcolemma, CaMKII has been demonstrated to tightly regulate SAN function and HR at the intracellular level (reviewed by Yaniv and Maltsev^5^). Mice treated with the CaMKII inhibitors AIP or KN-93 exhibit reduced HR, and ample additional evidence (reviewed by Wu and Anderson^6^) suggests CaMKII is a key regulatory protein of HR in addition to I_f_. NGS-based whole mRNA sequencing provided a comprehensive analysis suggestive of additional causative relationships between expressional changes and electrophysiological phenotype: (3) *Sema3a*, which was significantly downregulated in this analysis, was previously demonstrated to increase HR with cardiomyocyte-specific overexpression and to reduce HR in general knockout mice^7^; (4) *Cacna2d2*, encoding the L-type calcium channel subunit α2δ2, was downregulated. *Cacna2d2*^*−/−*^ mice exhibit lower HRs^8^. (5) *Scn10a* upregulation may contribute to lower HR, as mice expressing a hypermorphic Na_v_1.8 mutant exhibit reduced HR^9^. *SCN10A* encodes Na_v_1.8, a tetrodotoxin -resistant sodium channel originally identified in neurons^10^,^11^. It is also expressed in human heart tissue^11^ and highly enriched in cardiomyocytes of the conduction system^12^. However, it is important to note that genetic models of *Sema3A*^*−/−*^, *Cacna2d2*^*−/−*^, and *Scn10a* (hypermorphic mutation) were not cardiomyocyte specific, allowing for the possibility of neuronal effects on HR due to expression in both cell types. Whether via neuronal or cardiomyocytic effects, GWAS identified variants in *SCN10A* strongly associated with PR and QRS interval duration^11^,^12^,^13^. *Scn10a*^*−/−*^ mice exhibit shorter PR intervals^11^ and a gain-of-function single nucleotide polymorphism in *SCN10A* that prolonged PR interval was strongly associated with a decreased risk of ventricular fibrillation in the setting of acute myocardial infarction^11^, suggesting a beneficial role for higher SCN10A expression in cardiac electrophysiology. Another study in mice and rabbits examined the effects of SCN10A inhibition by A-803467 in isolated ventricular cardiomyocytes and demonstrated a reduction in late sodium current, shortened action potentials and suppressed arrhythmogenic early afterdepolarizations^14^. In our study, isolated cardiomyocytes from TG mice exhibited a markedly prolonged APD, which was fully reversible on SCN10A inhibition with A-803467. Importantly, APD in cardiomyocytes from *Scn10a*^*−/−*^ mice was insensitive to A-803467. This strongly suggests that APD prolongation in *TBC1D10C* TG mice is due to enhanced expression of SCN10A.

*TBC1D10C* TG mice displayed an increased median survival compared to WT controls, raising the question of the underlying mechanism. Reduced CaMKII and Ras activity may be cardioprotective. In addition, analysis of heart rate variability points to an altered processing of sympathetic activity. In theory, lower heart rate could result from reduced sympathetic stimulation of the sinus node, or result from a cellular level (less or altered beta receptors, altered downstream signal transduction, less cAMP, etc). Due to the cardiomyocyte-specific expression of TBC1D10C under control of the myosin heavy chain promoter and reduced contractile force and Ca^2+^ transients in isolated cardiomyocytes we strongly favor the latter possibility. Reduced susceptibility of cardiomyocytes to sympathetic signaling is a potential unifying mechanism explaining several of the observed alterations in TG mice, but further studies are necessary to identify the connection between TBC1D10C and sympathetic signal processing. Several long-term epidemiologic studies (e.g., the Framingham Study^15^, NHANES I Epidemiologic Follow-up Study^16^, Chicago studies^17^) demonstrated a strong correlation between elevated resting HR and increased incidence of both cardiovascular disease and all-cause mortality. Conversely, selective reduction of HR using the HCN4/I_f_ channel inhibitor ivabradine was shown to reduce clinical events in patients with heart failure^18^. In addition, mice treated with the I_f_ channel inhibitor ivabradine were recently demonstrated to have a significantly prolonged median lifespan^19^. This allows for the possibility that HR reduction per se is sufficient to increase median survival in our model. Mechanistically, the finding can be attributed to a more economic CO generation, that is, reduced cardiac ATP consumption per output and consequently decreased metabolic rate and oxidative stress, both being central mechanisms of cellular ageing. As a limitation of our study, we could not decipher whether these mechanisms are also sufficiently explaining enhanced exercise capacity during voluntary wheel running. Reduced Ras and CaMKII activity in our model allow for a plethora of posttranslational changes that warrant additional investigation.

Our data demonstrate a significant reduction of nuclear NFAT localization and hypertrophic growth response on acute overexpression of TBC1D10C in isolated cardiomyocytes. Therefore, we also investigated a potential antihypertrophic effect *in vivo*. Recently, Bisserier *et al.* reported attenuated hypertrophy in mice after retro-orbital sinus injection of AAV9 encoding TBC1D10C^20^ and Zhu *et al.* reported a near perfect rescue of TAC induced hypertrophy with TBC1D10C transgenic mice^21^. In contrast, our study with *TBC1D10C* TG mice did not display markedly blunted hypertrophy after TAC or chronic AngII infusion and only minor differences in myocyte diameters in myocardial infarction and 12 month old mice. Although *TBC1D10C* TG mice displayed reduced Ras activity, there was no robust reduction of CaN activity. The latter is in contrast to the findings of Bisserier and Zhu and offers an explanation for the lack of the anticipated large antihypertrophic effect in *TBC1D10C* TG mice. It is possible that these groups found a more ideal balance between pro-hypertrophic stimulus strength, timing and TBC1D10C activity to prevent hypertrophy. In addition, timeline of enhanced TBC1D10C expression may explain discrepant signalling and phenotype, including the possibility that compensatory signalling during long-term expression may antagonize some of the alterations observed by Bisserier *et al.* in more acute (AAV-9 mediated) overexpression. Furthermore, the aforementioned studies utilized C57BL/6 mice, whereas we analysed FVB/N transgenic mice. Our study underlines that impeded CaMKII and Ras (but not CaN) activities are robust findings on moderately long term enhanced TBC1D10C expression *in vivo*.

In summary, we demonstrate that enhanced cardiomyocyte specific expression of a single mRNA, *TBC1D10C*, reduces HR via expressional changes in *Hcn4*, *Scn10a*, *Sema3a*, and *Cacna2d2*, and reduced CaMKII and Ras activity. Mechanistically consistent with reduced HR and protection from cardiac stress signalling due to impeded sympathetic signal processing, CaMKII and Ras activity, TBC1D10C also enhances lifespan. TBC1D10C may be a new target for cardiac protection and longevity.

## Methods

Please refer to the online methods supplement for detailed description on next generation RNA sequencing, histological analysis, electrophysiology/ patch-clamp experiments, isolation and culture of cardiomyocytes, adenoviral gene transfer, real-time RT-PCR, heart rate variability analysis and gene ontology/pathway network analysis.

### Mice

The generation of *TBC1D10C* TG mice was performed by ligating human *TBC1D10C* cDNA with an N-terminal FLAG-tag sequence (the same TBC1D10C-Flag sequence construct was also used to generate adenovirus encoding TBC1D10C) and into a α-myosin heavy chain promoter-containing vector for cardiomyocyte selective expression. The alpha myosin heavy chain promoter was described earlier^22^. After purification and sequence confirmation, the construct was microinjected into the pronucleus of FVB/N mouse oocytes and implanted into pseudopregnant subjects according to standard techniques. Littermates were used as controls throughout the study. Adult mice hearts were isolated following sacrificing the mice by anaesthetic overdose with isoflurane (Abbott, Germany) and subsequent cervical dislocation. For Kaplan-Meier analysis of survival, only staff blinded towards the genotype had access to the animals. The principal endpoint was age at death (for mice found dead at daily inspections) or age at euthanasia (for severe sickness). Mice were excluded from the analysis (censored) at the indicated timepoint in the curve when subjected to scheduled health control, breeding or experimental analysis.

### Transmitter implantation and electrocardiography (ECG) monitoring

Perioperative analgesia was provided by addition of metamizole to the drinking water (1.33 mg/mL) from 2 days before until 2 days after surgery. In addition, 1 h before and immediately after surgery, buprenorphine (0.06 μg/mg body weight) was injected subcutaneously. Mice were anaesthetized with 2% isoflurane in O_2_. During anesthesia, a heating pad was used to maintain body temperature. ECG transmitter implantation was performed as described in detail elsewhere^23^. In brief, a 1.5-cm horizontal incision was made in the skin of the neck and a subcutaneous pocket was created. An ECG transmitter (Data Science International) was implanted in the pocket. Small incisions were made below the right scapula and in the apex area of the left chest, and a trocar was used to place ECG leads in lead II configuration. The tip of the leads was sutured to the chest wall and skin incisions were then closed. Continuous ECG recordings for 24 h were performed at least 72 h after surgery in awake, ambulatory mice using Dataquest software (Data Science International). An examiner blinded to group assignment then analysed data using ECG auto software (emka TECHNOLOGIES). QT-interval was corrected for heart rate using the formula QT_c_ = QT_o_/(R-R_o_/100)^1/2^. Further details are provided in the supplementary methods section.

### Running wheel exercise

Mice were housed in individual cages and had constant access to running wheels for 5 weeks. We recorded wheel revolutions continuously with a resolution of 1/16 revolution and a sampling rate of 1/3.75 s using LabVIEW™-based (National Instruments Corporation) custom software. Daily running duration, distance, and maximum velocity were calculated using a custom-designed MatLab^®^ program (MathWorks, Inc.) with the examiner blinded towards the genotype. The highest sample within 24 hours was set to maximum running velocity (Vmax). Data are presented as mean values of weeks.

### Transverse aortic constriction (TAC)

TAC surgery was carried as previously described^24^ with the surgeon blinded to the genotype. In brief, mice were anaesthetised with ketamine and xylazine (100 mg/kg and 5 mg/kg, i.p.). Spontaneously breathing mice (8 weeks old) were subjected to aortic ligation via a suprasternal access avoiding injury of the pleural cavity. A 27 gauge needle with a 5–0 suture was used to standardize constriction of the transversal aorta. Sham-operated animals were subjected to the same procedure including mobilization of the aortic arch but without ligation.

### Ras and CaN activation assay

Mice were injected with phenylephrine (10 mg/kg) 15 min before heart dissection. The Ras activation assay (Merck Millipore) was used according to the manufacturer’s protocol. In brief, left ventricles were homogenized in lysis buffer and lysates were centrifuged for 5 min at 14,000 *g*. Four milligrams of total protein were used for GTP-Ras pulldown with GST-(Raf1-) Ras binding domain beads. Beads were washed three times with cold lysis buffer and bound protein was eluted for 5 min at 95 °C with 2x Laemmli sample buffer. CaN activation assay (Enzo Life Sciences) was performed according to the manufacturer’s protocol.

### Western blot

The primary antibodies used were anti-GAPDH (Millipore), anti-Carabin (ProSci Inc.), anti-phospho-mitogen-activated protein kinase kinase 1/2 (MEK1/2) (Cell Signaling), anti-MEK1/2 (Cell Signaling), anti-phospho-CaM KinaseII pThr286 (Thermo Scientific), and anti-CaM KinaseII. Subsequently the Western blot membranes were incubated with a horseradish peroxidase (HRP)–coupled secondary antibody (GE Healthcare). Visualization was achieved by applying Supersignal West Pico- (Thermo Scientific) or Immobilon Western- (Millipore) chemiluminescence HRP substrate. Finally, image acquisition and densitometry were performed using Alphaview software (Alpha Innotech).

### RNA sequencing

Next generation sequencing (NGS)/RNA sequencing service using left ventricular RNA (WT vs. TG) was provided at the Transcriptome and Genome Analysis Laboratory in Göttingen and performed as detailed in Supplementary material online.

### Isolation and culture of cardiomyocytes and adenoviral gene transfer

We generated adenoviruses by ligation of cDNA coding sequence of *TBC1D10C* with an N-terminal FLAG-tag sequence, constitutively active Calcineurin A (CnA) or β-galactosidase (*LacZ*) into vector pDC515 and by flippase-mediated recombination with pBHGfrtΔE1,3FLP (Admax, Microbix) in HEK293 cells. An adenovirus expressing NFATc3 as a fusion protein with green fluorescent protein (GFP) (NFATc3-GFP) was kindly provided by R. Marchase. The adenoviruses were then purified via cesium chloride gradient centrifugation. Neonatal ventricular rat cardiomyocytes were isolated from 1- to 2-day old Wistar rats as described previously^25^ and detailed in Supplementary material online. In brief, neonatal rats were sacrificed by decapitation and hearts were quickly dissected. Ventricles were minced in phosphate-buffered saline (PBS) containing 0.2% (w/v) trypsin (Biochrom AG) and 0.1% (w/v) collagenase type II (Worthington).

### Statistics

Unless stated otherwise in the figure legend, statistical analysis was performed using Prism software version 5.01 (Graphpad) with a two-tailed unpaired Student’s t-test or a one-way ANOVA with Bonferroni posttest correction where appropriate. Survival fractions were calculated using the product limit (Kaplan-Meier) method with log-rank test. Graphs represent mean ± standard error. A *P* < 0.05 was considered statistically significant.

### Study approval

All animal experiments conformed to the guidelines from Directive 2010/63/EU of the European Parliament and were approved by the Animal Ethics Committee of the University of Göttingen and the German federal state government.

## Additional Information

**How to cite this article**: Volland, C. *et al.* Enhanced cardiac *TBC1D10C* expression lowers heart rate and enhances exercise capacity and survival. *Sci. Rep.* **6**, 33853; doi: 10.1038/srep33853 (2016).

## Supplementary Material

Supplementary Information
