# Supplementary material for: Enhanced cardiac TBC1D10C expression lowers heart rate and enhances exercise capacity and survival
Source: Sci Rep. 2016 Sep 26;6:33853. doi: 10.1038/srep33853 (PMC5036039; doi:10.1038/srep33853)

Online Supplement to

Enhanced cardiac *TBC1D10C* expression lowers heart rate and enhances exercise capacity and survival

Cornelia Volland[1], Sebastian Bremer, Kristian Hellenkamp, Nico Hartmann, Nataliya Dybkova, Sara Khadjeh, Anna Kutschenko, David Liebetanz, Stefan Wagner, Bernhard Unsöld, Michael Didié, Karl Toischer, Samuel Sossalla, Gerd Hasenfuß, & Tim Seidler

Content:

Expanded methods

Supplemental References

Supplemental Figure Legends

Supplemental Table 1

Supplemental Figure 1

Supplemental Figure 2

Supplemental Figure 3

Supplemental Figure 4

Supplemental Figure 5

Supplemental Figure 6

Expanded methods

Histological analysis

After anaesthetic overdose with isoflurane (Abbott, Germany), mice were humanely killed by cervical dislocation. Hearts were quickly excised and fixed with 4% (w/v) paraformaldehyde in PBS. Paraffin-embedded 6-µm sections were stained with wheat-germ-agglutinin (WGA) tetramethyl rhodamine isothiocyanate (TRITC)-coupled lectin (Sigma-Aldrich). An examiner blinded towards the group assignment measured myocyte diameter using Axio Vision (Zeiss) and ImageJ software. We examined identical areas of the left mid-ventricular walls. Myocyte diameters were measured on transverse sections. At least 100 transverse cardiomyocyte diameters per mouse were examined. For Picrosirius Red staining dewaxed and hydrated sections were stained with 1% (w/v) Sirius red (Sigma Aldrich) in saturated aqueous solution of picric acid (Sigma Aldrich) for 1 hour.

Real-time RT-PCR

Total RNA was isolated from left ventricular or atrial tissue using the RNeasy fibrous tissue Mini Kit (Qiagen). Complementary DNA (cDNA) was synthesized from 200 ng of total RNA using an iScript cDNA synthesis Kit (Bio-Rad) and real-time RT-PCR was performed with the IQ SYBR Green Supermix and iCycler iQ Detection System (Bio-Rad). Starting quantities were extrapolated from standard curves for each primer set and normalized to 18S RNA levels. Primer sequences: 18S-for: 5' CGA AAG CAT TTG CCA AGA AT3'; 18S-rev: 5' GAG GTT TCC CTG GTT GAG TC3'; hTBC1D10C-for: 5' CCTGGTGCAGATCTGTGAGGT3', hTBC1D10C-rev: 5' AAGAGGCACAGGAACCACTCG3', MYH6-for: 5' CCG GGT GAT CTT CCA GCT AAA3', MYH6-rev: 5' GCT CAG CAC ATC AAA GGC ACT3', MYH7-for: 5' TCC CAA GGA GAG ACG ACT GTG3', MYH7-rev: 5' CCT TAA GCA GGT CGG CTG AGT3', BNP-for: 5' TCT CCA GAG CAA TTC AGA T3', BNP-rev: 5' AAC AAC TTC AGT GCG TTA CA3', Pde1c-for: 5' CGA GCC AAG GTA CCC AAA GA3', Pde1c-rev: 5' ATC ACC CTT GCG TGT TCC AT3', Sema3a-for: 5' CCC ACT GAC TCA CTG CTC TG3', Sema3a-rev: 5' GGC TCT CTG TGA CTT CGG AC3', MmScn10a_1_SG: QuantiTect Primer Assay QIAGEN #QT01076740 (Qiagen), Mm_HCN4_1_SG: QuantiTect Primer Assay QIAGEN #QT00268660 (Qiagen).

Adult cardiomyocyte isolation, Ca transient and shortening measurements and patch-clamp experiments

For cell shortening and Ca transient measurements, left ventricular cardiomyocytes were freshly isolated from hearts of adult WT or TG littermates in Langendorff mode. Myocytes were loaded with fluo-3 AM (10 µmol/l) for 15 min. After loading, cells were washed with Tyrode's solution (in mmol/L) 140 mmol/l NaCl, 4 mmol/l KCl, 1 mmol/l MgCl, 5 mmol/l HEPES, 10 mmol/l glucose, 1 mmol/l $CaCl_2$, pH=7.4. Myocytes were field-stimulated (voltage 25% above the threshold, 1 Hz, 37Cº). Shortening and Ca transients were measured simultaneously (Ion-optix, Milton, Massachusetts). Stimulation frequency was increased stepwise from 1 to 4 Hz.

We isolated single atrial cardiac myocytes from adult mouse hearts. Mice were anaesthetized with isoflurane (1.5% v/v in 100% O_2) (Abbott, Germany). Afterwards, hearts were carefully excised and we used a Langendorff apparatus to perfuse explanted mouse hearts retrogradely with an initially Ca^{2+}-free Tyrode's solution containing: 113 mmol/L NaCl, 4.7 mmol/L KCl, 0.6 mmol/L KH_2PO_4, 0.6 mmol/L $Na_2HPO_4{\cdot}2H_2O$, 1.2 mmol/L $MgSO_4{\cdot}7H_2O$, 12 mmol/L $NaHCO_3$, 10 mmol/L $KHCO_3$, 10 mmol/L HEPES, 30 mmol/L taurine, 10 mmol/L 2,3-butanedione monoxime, 5.5 mmol/L glucose, 0.032 mmol/L phenol red (37°C, pH 7.4). Then, 0.075 mg/mL of Liberase TM (Roche Diagnostics, Mannheim, Germany) and 0.014% trypsin as well as 0.125 mmol/L of $CaCl_2$ were added to the perfusion solution. After the extracellular matrix was digested up to the point of visible tissue solution, the heart was transferred to a glass basin and the atria were carefully separated from the ventricles. Subsequently, the atria were cut with preparatory scissors into small pieces of tissue. After preparation of the mouse hearts Ca^{2+} reintroduction was performed carefully through stepwise increasing [Ca^{2+}] from 0.1 to 1.6 mmol/L.

For action potential recordings, a ruptured-patch whole-cell voltage-clamp was used to measure membrane potential (current clamp configuration). For membrane potential measurements, microelectrodes were filled with 92 mmol/L K-aspartate, 48 mmol/L KCl, 1 mmol/L Mg-ATP, 10 mmol/L HEPES, 0.02 mmol/L EGTA, 0.1 mmol/L GTP-Tris, and 4 mmol/L Na_2-ATP (pH 7.2, KOH). The bath solution contained 140 mmol/L NaCl, 4 mmol/L KCl, 1 mmol/L $MgCl_2$, 2 mmol/L $CaCl_2$, 10 mmol/L glucose, and 10 mmol/L HEPES (pH 7.4, NaOH). Access resistance was typically ca. 5–15 MΩ after patch rupture. Action potentials were continuously elicited by square current pulses of 1–2 nA amplitude and 1–5 ms duration at increasing stimulation frequencies (0.5–3 Hz). When appropriate, A-803467 (30 nmol/L) vs. vehicle control was added to the bath solution. Fast capacitance was compensated in cell-attached configuration. Membrane capacitance and series resistance were compensated after patch rupture. Signals were filtered with 2.9- and 10-kHz Bessel filters and

recorded with an EPC10 amplifier (HEKA Elektronik). Recordings were started 5 min after rupture. All experiments were conducted at a temperature of 37.7°C by an examiner blinded towards the group assignment.

Isolation and culture of neonatal rat cardiomyocytes and adenoviral gene transfer

We generated adenoviruses by ligation of cDNA coding sequence of *TBC1D10C*, constitutively active Calcineurin A (CnA) or β-galactosidase (*LacZ)* into vector pDC515 and by flippase-mediated recombination with pBHGfrtΔE1,3FLP (Admax, Microbix) in HEK293 cells. An adenovirus expressing NFATc3 as a fusion protein with green fluorescent protein (GFP) (NFATc3-GFP) was kindly provided by R. Marchase. The adenoviruses were then purified via cesium chloride gradient centrifugation. Neonatal ventricular rat cardiomyocytes were isolated from 1- to 2-day old Wistar rats. Neonatal rats were sacrificed by decapitation and hearts were quickly dissected. Ventricles were minced in phosphate-buffered saline (PBS) containing 0.2% (w/v) trypsin (Biochrom AG) and 0.1% (w/v) collagenase type II (Worthington). Four digestion cycles were performed to dissociate the cells. Cells were centrifuged and resuspended in DMEM:F12 medium with 10% (v/v) fetal calf serum and pre-plated onto tissue culture dishes. Finally, myocytes were plated on gelatine-coated tissue culture dishes, switched to serum-free medium 16 h after plating and transfected with adenovirus.

Planimetric measurements and α-actinin staining

To examine cellular hypertrophy, adenoviral gene transfer was performed in neonatal ventricular rat cardiomyocytes. The cells were incubated with adenovirus at the indicated multiplicity of infection (MOI) and treated with 5 µM phenylephrine, 10 nM AngII (Sigma-Aldrich) or vehicle as a control. After 48 h, myocytes were fixed, permeabilized with 0.1% (v/v) Triton X-100 in PBS and blocked with 4% bovine serum albumin (w/v) in PBS.

Staining was conducted with a monoclonal mouse α-actinin antibody (Sigma) followed by Cy3-coupled anti-mouse antibody (Dianova) and 4´,6-diamidino-2-phenylindole (DAPI) for nuclei visualization. Cell surface areas were determined using Axio Vision and ImageJ software (Zeiss). The analysis was performed by an examiner blinded towards the group assignment.

Echocardiography

Mice were anaesthetized with 1.5% isoflurane (v/v in 100% O_2) (Abbott, Germany) and echocardiography was performed using the Vevo 2100 (Figure 1 and 2, Suppl. Figure 5b) or 600 (Figure 3, Suppl. Figure 5e) system (Visualsonics). M-mode images were obtained to determine wall dimensions (and FS in Fig. 3 and Suppl. Figure 5e) by adjusting the M-Mode line 90° to the septal endocardial border in the parasternal long-axis view at the mid-papillary level with the section adjusted to obtain maximum end-diastolic left ventricular diameter. B-mode images were recorded from the short- and long-axis views for determination of fractional area shortening (FAS) and ejection fraction (EF) at the mid-papillary levels (Fig. 1 and 2). The examiners were blinded towards the genotype and group assignment.

RNA sequencing

NGS was performed at the Transcriptome and Genome Analysis Laboratory core facility in Göttingen. RNA sequencing was applied for WT (n=11) and TG (n=9) . Full RNA was isolated from left ventricular tissue using Qiazol (Qiagen) according to the manufacturer`s protocol, followed by additional purification with miRNeasy Mini spin columns (Qiagen). We started with 1 µg of total RNA and performed the library preparation according to the instructions in the TruSeq RNA Sample Preparation v2 Kit from Illumina (Cat. N°RS-122-2002). For accurate quantitation of cDNA libraries we used the fluorometric based QuantiFluor™ dsDNA System from Promega (Mannheim, Germany). The size of final cDNA libraries was determined using the DNA 1000 chip on the Bioanalyzer 2100 from Agilent (280 bp). cDNA libraries were amplified and sequenced by using the cBot and HiSeq2000 from Illumina (SR; 1x50 bp; ca. 30 Mio reads per sample).
Sequence images were transformed to bcl files with Illumina software BaseCaller, and then demultiplexed to fastq files with CASAVA v1.8.2. Quality checking was done via fastqc. Sequences were aligned to the genome reference sequence of *Mus musculus* (GRCm38/mm10). Alignment was performed using the STAR alignment software (version 2.3.0e) [1] allowing for two mismatches within 50 bases. Subsequently, conversion of resulting SAM files to sorted BAM files, filtering of unique hits and counting were conducted with SAMtools (version 0.1.19)[2] and HTSeq (version 0.6.1p1) [3]. Data were preprocessed and analysed in the R/Bioconductor environment (www.bioconductor.org) using the DESeq2 package (version 1.8) [4]. Specifically, the data were normalized and tested for differentially expressed genes based on a generalized linear model likelihood ratio test assuming negative binomial data distribution. Candidate genes were filtered to a minimum of 20% ($\log_2(0.25)$-

fold) change and false discovery rate–corrected *P*-value < 0.05. Gene annotation was performed using *Mus musculus* entries from Ensembl (www.ensembl.org) via the biomaRt package (version 2.18.0) [5].

Myocardial infarction in mice

Permanent ligation of the LAD immediately below the left atrial appendage was performed under isoflurane anaesthesia as detailed in [6]. In brief, a left lateral thoracotomy was performed to expose the heart. Following pericardiotomy the LAD was visualized and ligated with a 9-0 prolene suture with an atraumatic needle. Successful ligation was confirmed by bleaching and hypokinesis of the perfused myocardium.

Telemetric ECG and analysis of heart rate variability

Telemetry was conducted in conscious mice. ECG (sampling frequency 1000 Hz) and motor activity (sampling frequency 100 Hz) were measured with subcutaneously implanted transducers (Data Science International). Mice were kept in individual cages with 12h/12h light/dark cycle. In order to achieve an equal treatment of mice of both groups, transmitter surgery was always done in a pair of littermates including one transgenic and one wild-type mouse. Continuous ECG recordings were then started simultaneously in one pair of littermates. An examiner blinded to group assignment then analysed data offline. Heart rate was calculated from the interval between two consecutive R waves of ECG over the whole 24 hours period (thus including activity and inactivity periods).

Time-domain analysis of heart rate variability was analyzed for 24h after automatic exclusion of ectopic beats. Ectopic beats were defined as R-R values not contained between mean R-R interval plusminus two times standard deviation (95.5% confidence intervals). Telemetry recordings were excluded from analysis if the number of abnormal/ectopic beats exceeded 5% of the total beats.

The time-domain parameters calculated over 24h were: mean R-R interval (NN, ms), standard deviation of all normal R-R intervals (SDNN, ms), square root of the mean square successive differences between successive normal intervals (RMSSD, ms) reflecting short-term variations in heart rate, percentage of normal consecutive R-R intervals differing by more than 6 ms (pNN6 in %), and coefficient of variation CV (%) defined as SDNN/NN*100.

Frequency-domain analysis was conducted for 3 minute short intervals every 2 hours in phases without motor activity and in segments without ectopic beats and artifacts. During a 24 h period 12 analysis (segment length 2048 beats, linear interpolation and resampling to a 20 Hz interbeat time series, von-Hann windowing) were averaged. Cut-off frequencies for low-frequency (LF) were 0.15-1.5 Hz and for high-frequency (HF) 1.5-5 Hz. Data was reported as absolute power (ms2) and in normalized units (nu). Normalization for LF and HF were done by LF/(total power-very low frequency)*100 and HF/(total power-very low frequency)*100, respectively.

Gene ontology and pathway enrichment analysis

The analysis is based on gene ontology (GO) molecular function and reactome databases. GO and pathway enrichment analysis was conducted using the Cytoscape plug-in ClueGO software [7]. The analysis was fed with significantly regulated genes (cut off adjusted p-value<0.05) from the transcriptome of WT mice compared to TG mice. The created network represents GO/functional terms as nodes, which are linked based on a predefined kappa score level. The size of the nodes reflects the enrichment significance of the terms. The functional groups are created by iterative merging of initially defined groups based on the predefined kappa score threshold.

Supplemental References

1 Dobin, A. *et al.* STAR: ultrafast universal RNA-seq aligner. *Bioinformatics* **29**, 15-21 (2013).

2 Li, H. *et al.* The sequence alignment/map format and SAMtools. *Bioinformatics* **25**, 2078-2079 (2009).

3 Anders, S., Pyl, P. T. & Huber, W. HTSeq--A Python framework to work with high-throughput sequencing data. *Bioinformatics*, btu638 (2015).

4 Love, M. I., Huber, W. & Anders, S. Moderated estimation of fold change and dispersion for RNA-seq data with DESeq2. *Genome Biol* **15**, 550 (2014).

5 Durinck, S. *et al.* BioMart and Bioconductor: a powerful link between biological databases and microarray data analysis. *Bioinformatics* **21**, 3439-3440 (2005).

6 Unsold, B. *et al.* Melusin protects from cardiac rupture and improves functional remodelling after myocardial infarction. *Cardiovasc Res* **101**, 97-107, doi:10.1093/cvr/cvt235 (2014).

7 Bindea, G. *et al.* ClueGO: a Cytoscape plug-in to decipher functionally grouped gene ontology and pathway annotation networks. *Bioinformatics* **25**, 1091-1093, doi:10.1093/bioinformatics/btp101 (2009).

Supplemental Figure Legends

Supplemental Figure 1: Phenotype of *TBC1D10C* TG line 2.

(**a**) Real-time RT-PCR displaying significant higher transgenic *TBC1D10C* mRNA overexpression level in TG line 1 vs. TG line 2 (line 1: *n*=7, line 2: *n*=10 mice; *P*<0.001); Student's *t*-test. (SQ: template starting quantity.) (**b**) Western blot displaying significant higher TBC1D10C protein level in TG line 1 vs. TG line 2 (line 1: *n*=4, line 2: *n*=3 mice; *P*<0.05); Student's *t*-test. (**c**) Echocardiography in 11-week-old mice of line 2 (WT vs. TG) revealed significantly increased fractional area shortening (FAS) and ejection fraction (EF) and similar stroke volume (SV), heart rate (HR) and cardiac output (CO) in the TG. (WT: *n*=15, TG: *n*=15 mice; *P*<0.05; Student's *t*-test). (**d**) *Hcn4*, *Scn10a* and *Sema3a* expression levels in *TBC1D10C* TG line 2 were estimated by real-time RT PCR (WT: *n*=12, TG: n=8 mice; *P*<0.05; Student's *t*-test). (SQ: template starting quantity.)

Supplemental Figure 2: Analysis of CnA/NFAT signaling and hypertrophy in isolated cardiomyocytes.

(**a**) NFATc3-GFP localization in neonatal rat cardiomyocytes expressing constitutively active Calcineurin A (CnA) with cotransfection of TBC1D10C. Cells were transduced with adenovirus as detailed in the graph and nuclear NFAT-GFP localization was determined after 48 h. Scale bar: 10 µm (*n*=3 experiments; for each experiment 200 cells per group; *P*<0.05). (**b**) Relative cell surface area of α-actinin stained neonatal rat cardiomyocytes transduced with lacZ vs. TBC1D10C encoding adenovirus. TBC1D10C inhibited phenylephrine- and angiotensin II-induced hypertrophic cell growth (>100 cells per group, *n*=3 experiments; *P*<0.0001). Scale bar: 5 µm.

Supplemental Figure 3: Additional analysis of mice subjected to TAC

This Figure complements Fig.2 of the manuscript. 8-week-old *TBC1D10C* TG mice underwent TAC intervention with a 27G needle or were sham operated. Echocardiography 4 weeks after intervention revealed significantly increased diastolic anterior wall thickness (AWThd) in TAC vs. sham mice, but there was no difference between WT and TG. Left ventricular inner diameter (LVIDd) was not different, and fractional area shortening (FAS) and ejection fraction (EF) were increased in TG sham vs. WT sham but not after TAC. (n=9–15 mice per group; P<0.05) (scale bar: 1 mm).

Supplemental Figure 4: Effect of TBC1D10C in a model of myocardial infarction and in aged mice.

Eight week old mice underwent surgical ligation of the left anterior descending artery or sham surgery. (**a**) Morphometry 2 weeks after the myocardial infarction demonstrates higher heart weight in TAC vs. sham operated animals. Histological analysis (WGA stain) of unaffected regions of the LV exhibited slightly smaller increase in cross sectional diameter in TG vs. WT mice. (**b**) Echocardiography 2 weeks after surgery revealed significantly reduced left ventricular fractional area shortening (FAS) and increased end-diastolic inner diameter (LVIDD) in mice subjected to myocardial infarction vs. sham operated mice, but there was no difference between WT and TG. TG mice (both sham and MI) exhibited a lower heart rate compared to Wt MI mice. (n=8–11 mice per group; P<0.05 T-test). (**c**) Quantitative real-time RT PCR exhibits a marked myosin heavy chain isoform shift and increase in BNP expression due to myocardial infarction, but no significant differences between the genotypes. (**d**) In 12 month old mice, histological analysis with WGA revealed a small but statistically significant reduction in myocyte cross sectional diameter (left panel frequency histogram, right panel bar diagram). (**e**) Echocardiography revealed significantly lower heart rate and a numerical trend towards better fractional shortening (FS) (12 mice per group, P< 0.05 T-test). (**f**) There were no significant differences in fibrosis, as determined with Picrosirius red stain (scale bar: 50 µm).

Supplemental Figure 5: Network of significantly regulated genes in the transcriptome of Carabin TG mice compared to WT mice.

(**a**) The ClueGO analysis resulted in a gene ontology/pathway term network showing significant overlapping functional groups (p-value<0.05) of a number of regulated genes relevant to cardiac muscle contraction, cardiac conduction and the regulation of heart rate as well as the regulation of sodium ion transmembrane transport. The created network represents GO/functional terms as nodes, which are linked based on a predefined kappa score level. The size of the nodes reflects the enrichment significance of the terms. The functional groups are created by iterative merging of initially defined groups based on the predefined kappa score threshold. The final groups are colored (please find colored squares in figure for color code of the most relevant groups) and overlaid with the network. Functional groups are represented by their most significant (leading) term and are visualized in the network. pV= P-value. (**b**) Differential expression of genes of Carabin TG mice compared to the control and assigned to muscle contraction, cardiac conduction and sodium ion transport by ClueGO Analysis. The Graph shows the log2 fold change (log2FC) of expression of genes in TG mice compared to the control. Bars highlighted in blue are assigned to muscle contraction, green color refers to cardiac conduction and purple to sodium ion transport. All genes are significantly regulated (adjusted p-value <0.05). rpkm = reads per kilobase per million mapped reads.

Supplemental Figure 6: full length blot membranes corresponding to cropped blots.

These uncropped blot membranes correspond to the cropped blots in the figures of the manuscript as indicated. Line indicates cuts to allow for different antibody exposure.

Supplemental Table 1

	WT (n=6)	TG (n=6)
**Time-domain**		
**NN (ms)**	100.6±0.9	106.6±2.4*
**SDNN (ms)**	11.49±1.153	8.75±1.29 *
**RMSSD (ms)**	3.17±0.21	2.48±0.31, p=0.08 vs. WT
**NN6 (%)**	7.69±1.42	2.86±0.82*
**CV (%)**	11.39±1.11	8.14±1.14*
**Frequency-domain**		
**LF (ms2)**	2.18±0.31	0.48±0.13*
**HF (ms2)**	4.53±0.52	1.97±0.43*
**LF (nu)**	30.42±4.07	12.37±2.25*
**HF (nu)**	69.34±4.03	75.32±10.79
**LF/HF**	0.49±0.08	0.18±0.04*

Supplemental Table 1: Analysis of heart rate variability for wild-type (WT) vs. transgenic (TG) mice.

NN (mean R-R interval), **SDNN** (standard deviation of all normal R-R intervals), **RMSSD** (square root of the mean square successive differences between successive normal intervals), **NN6** (percentage of normal consecutive R-R intervals differing by more than 6 ms), CV (coefficient of variation defined as SDNN/NN*100); LF (low frequency) and HF (high frequency) were reported in absolute and normalized units (nu); *P<0.05 vs. WT; T-Test.

Supplemental Figure 1

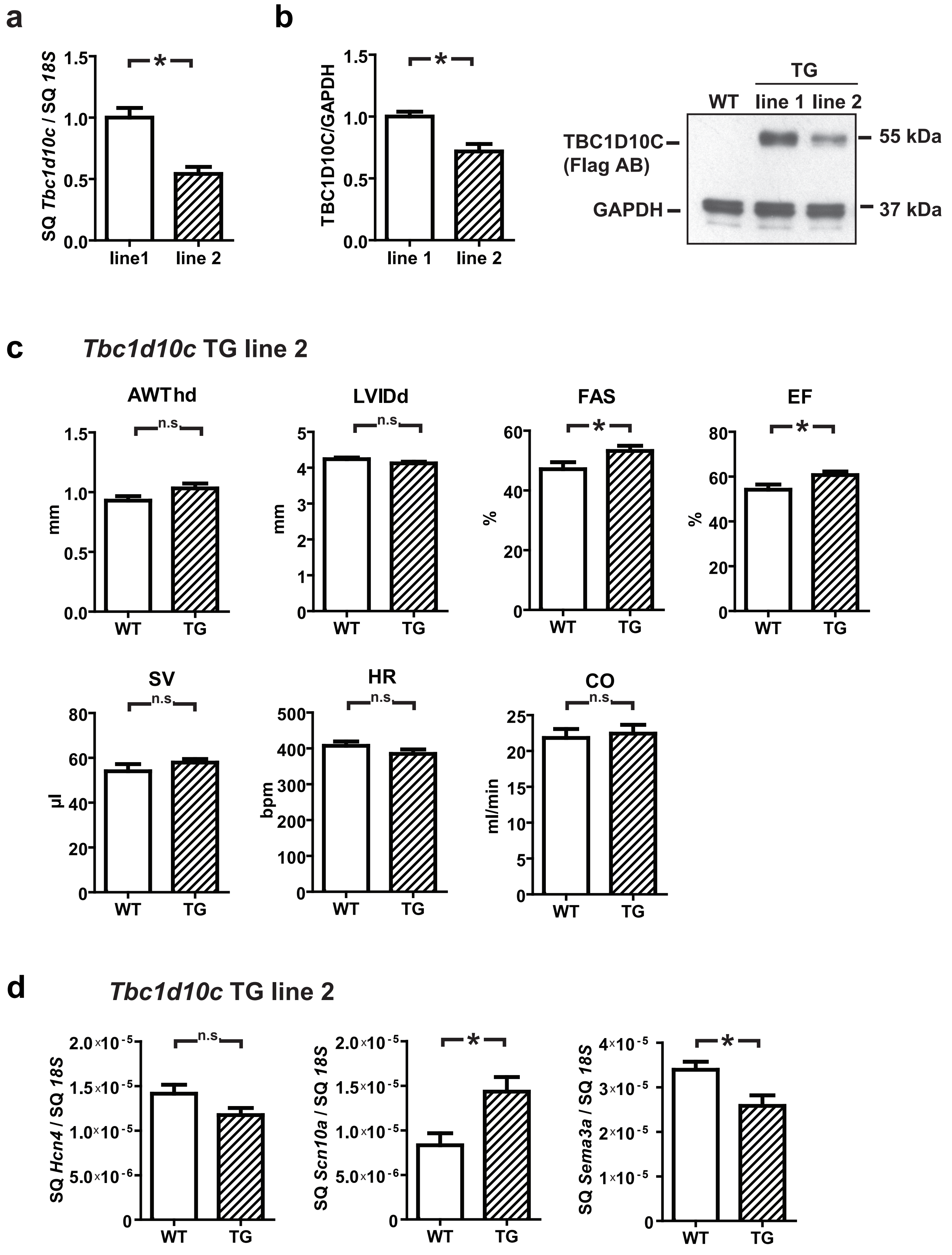

Supplemental Figure 2

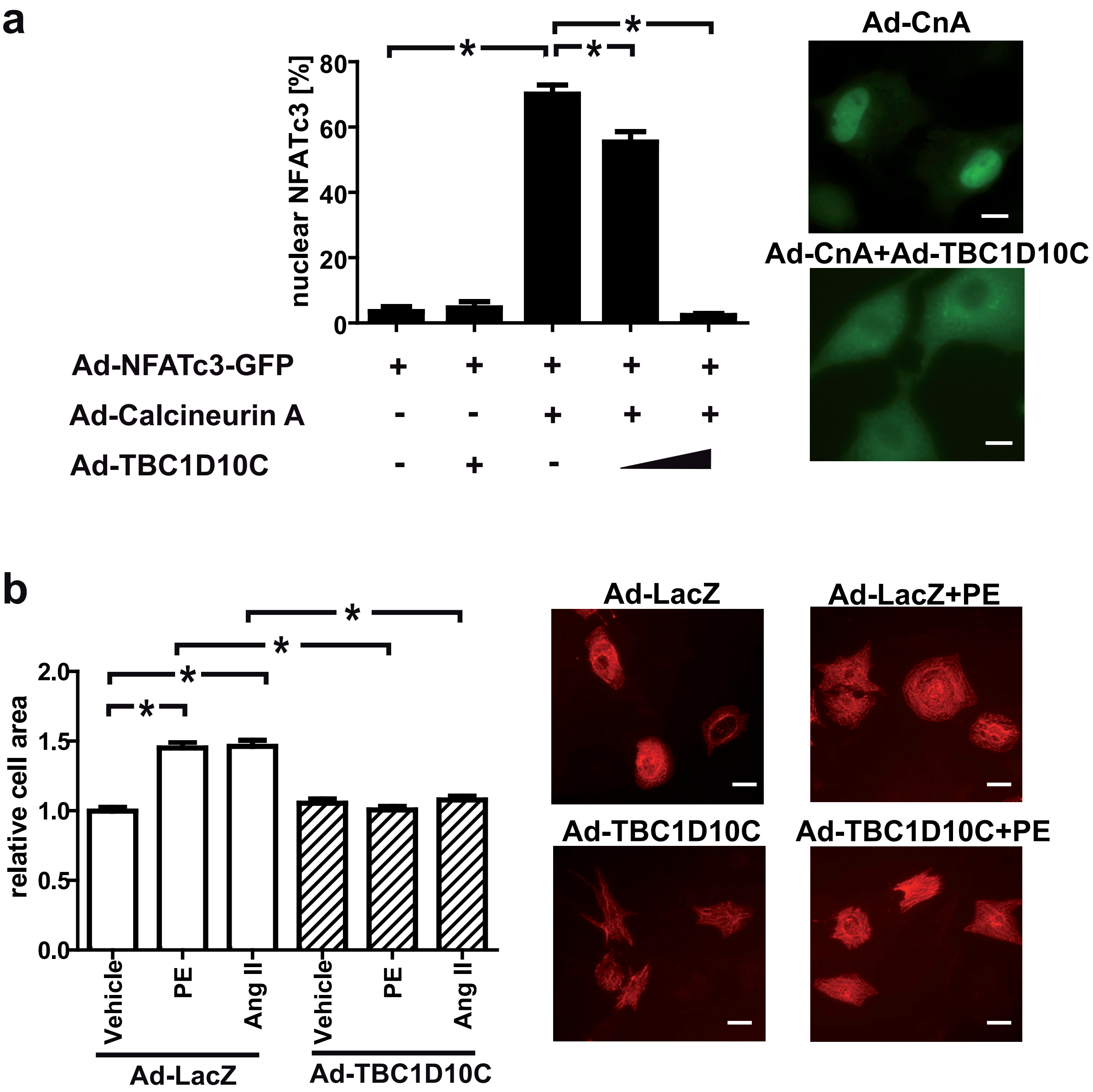

Supplemental Figure 3

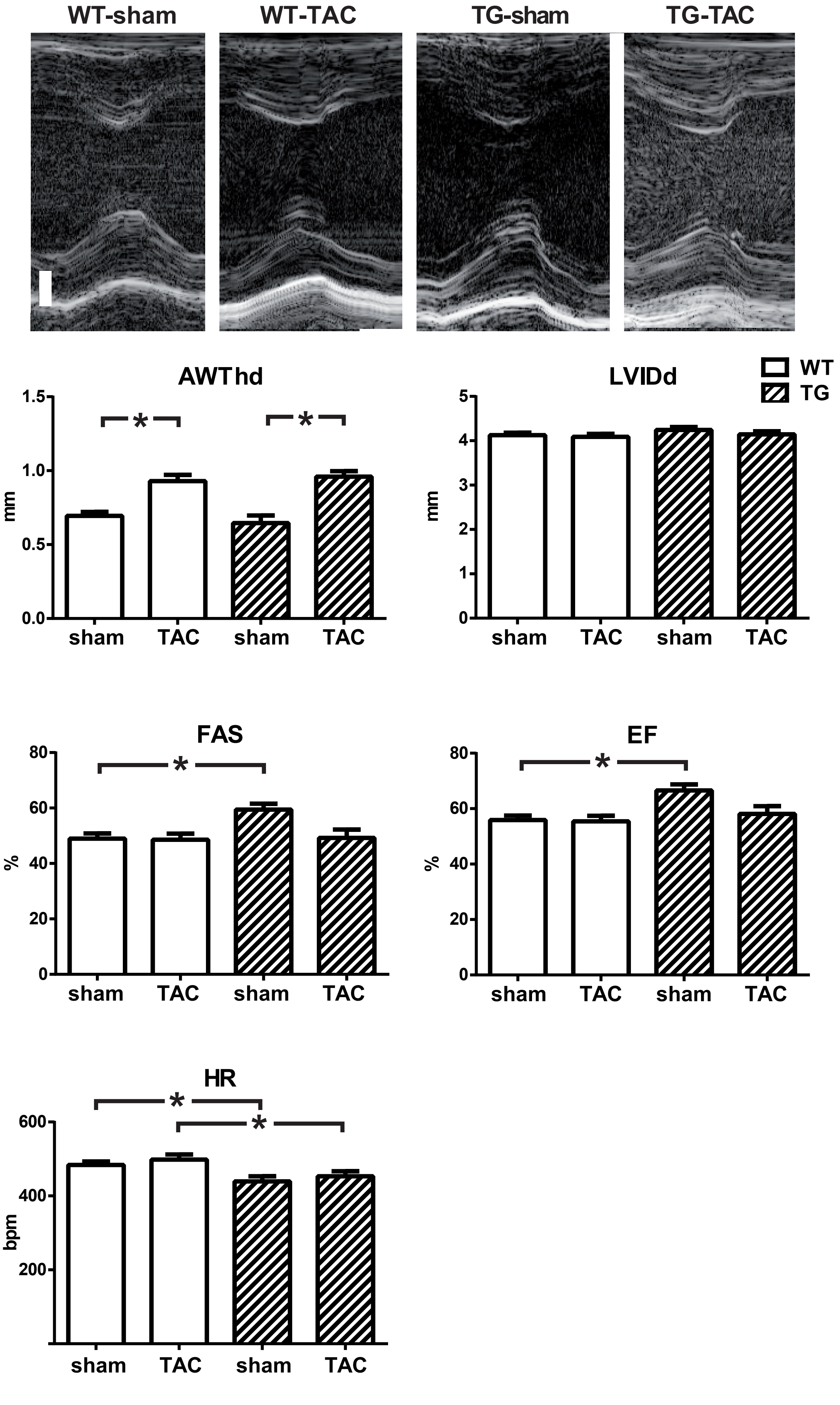

Supplemental Figure 4

Effect of TBC1D10C on hypertrophy in a model of myocardial infarction

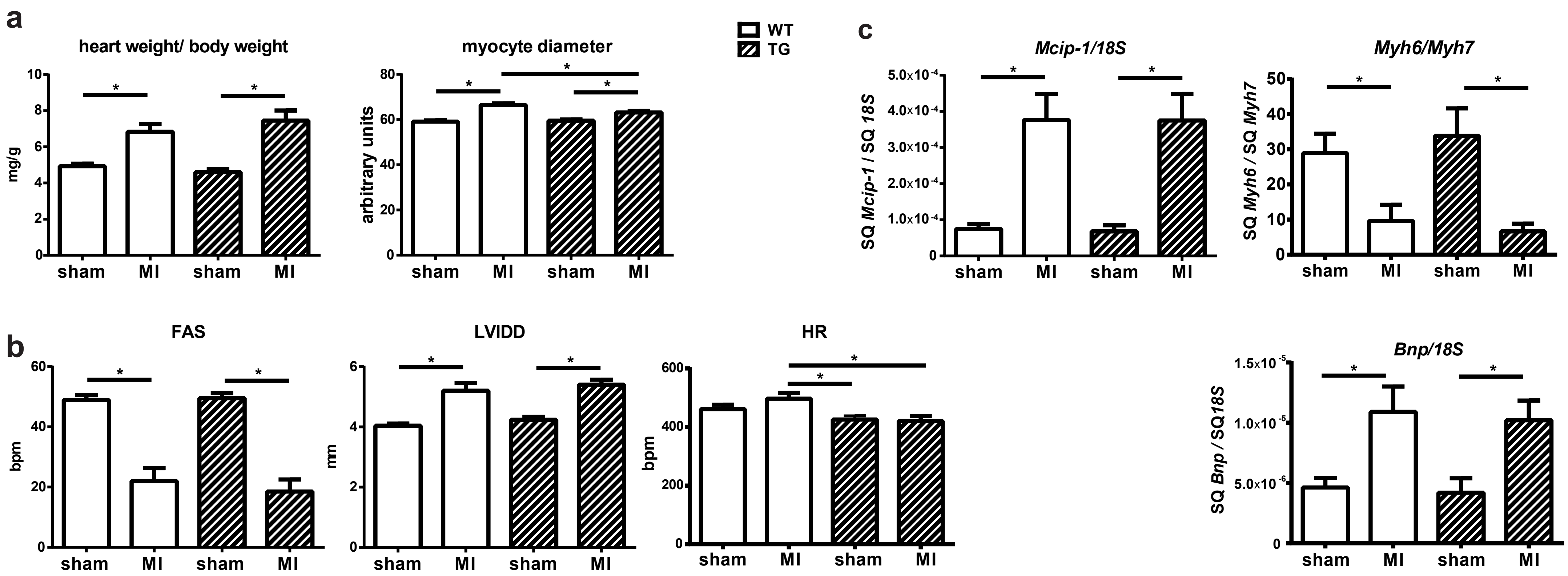

Effect of TBC1D10C in 12 month old mice

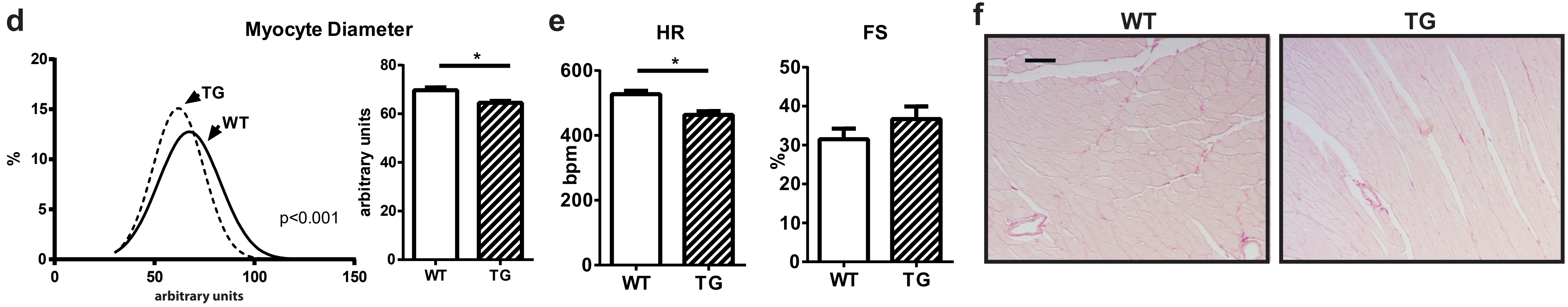

Supplemental Figure 5

a

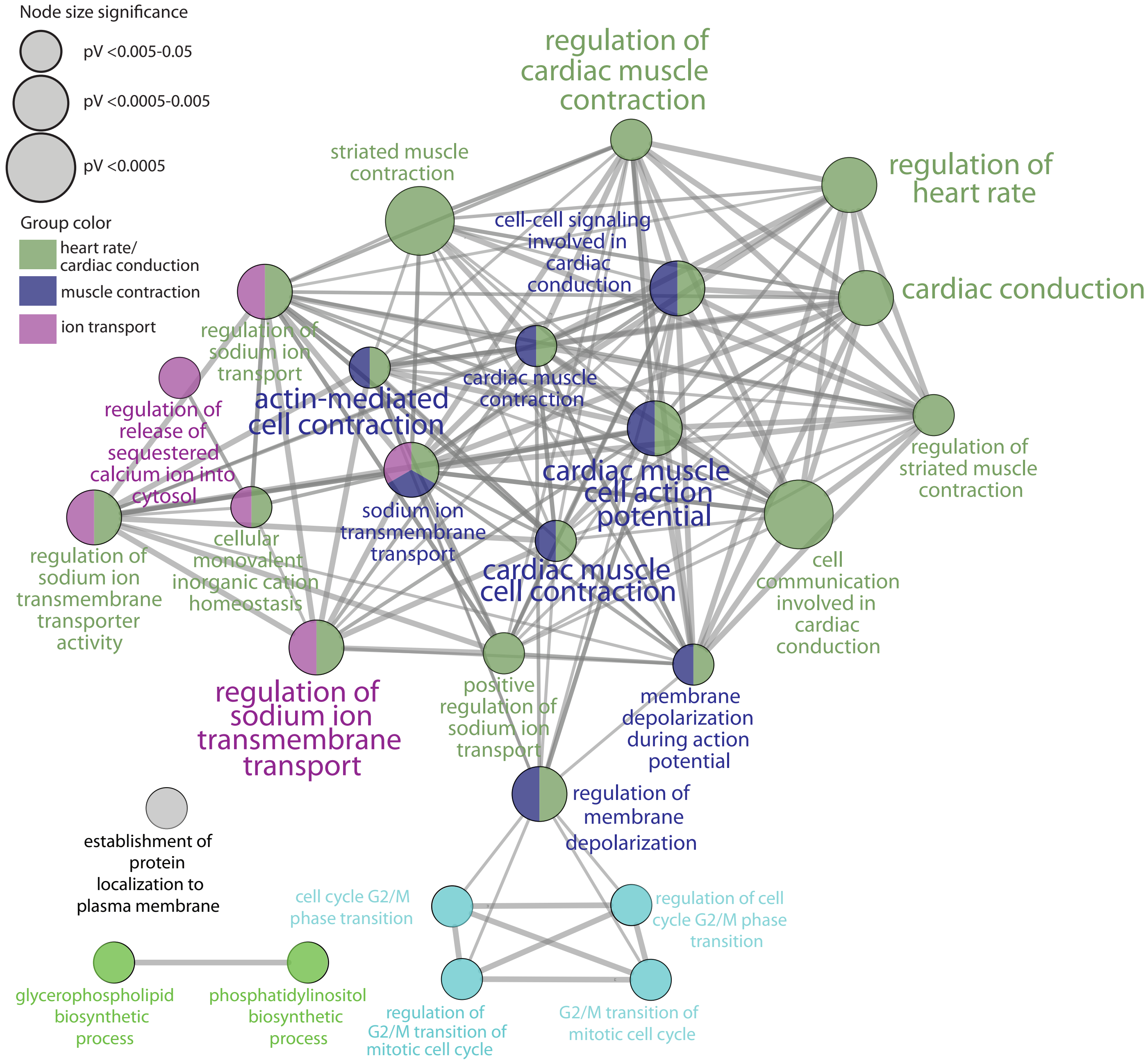

b

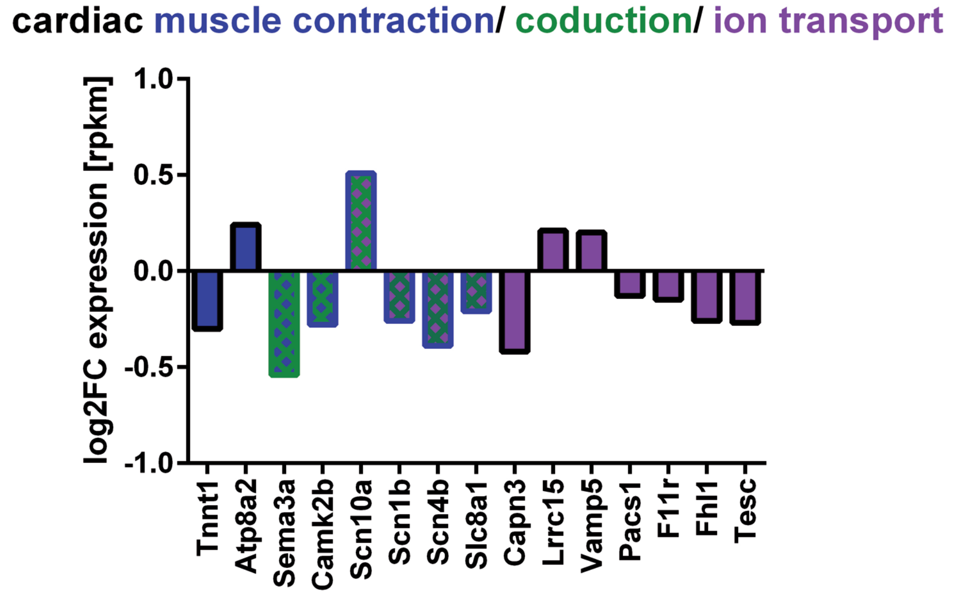

Supplemental Figure 6

Figure 1 a

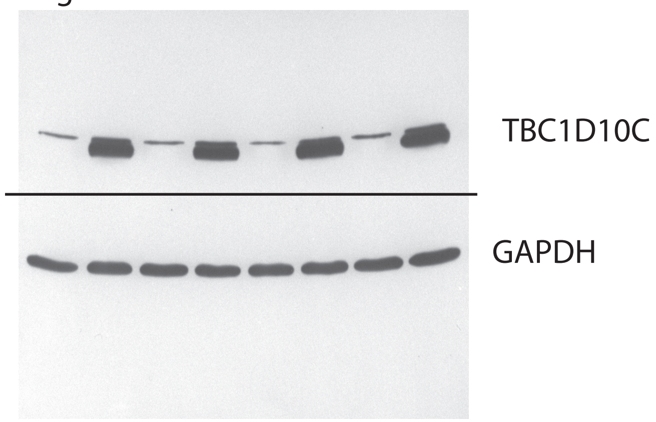

Figure 4b

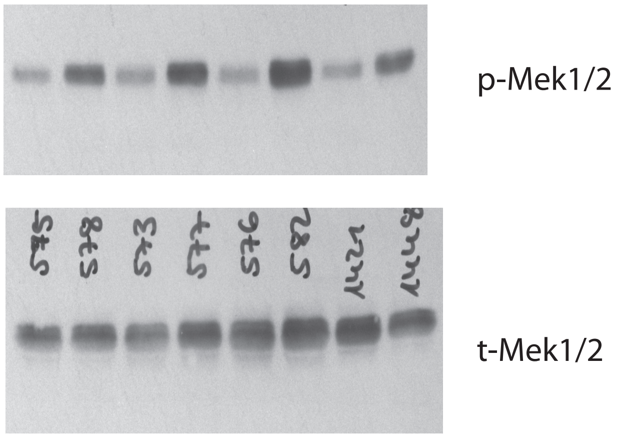

Figure 4e

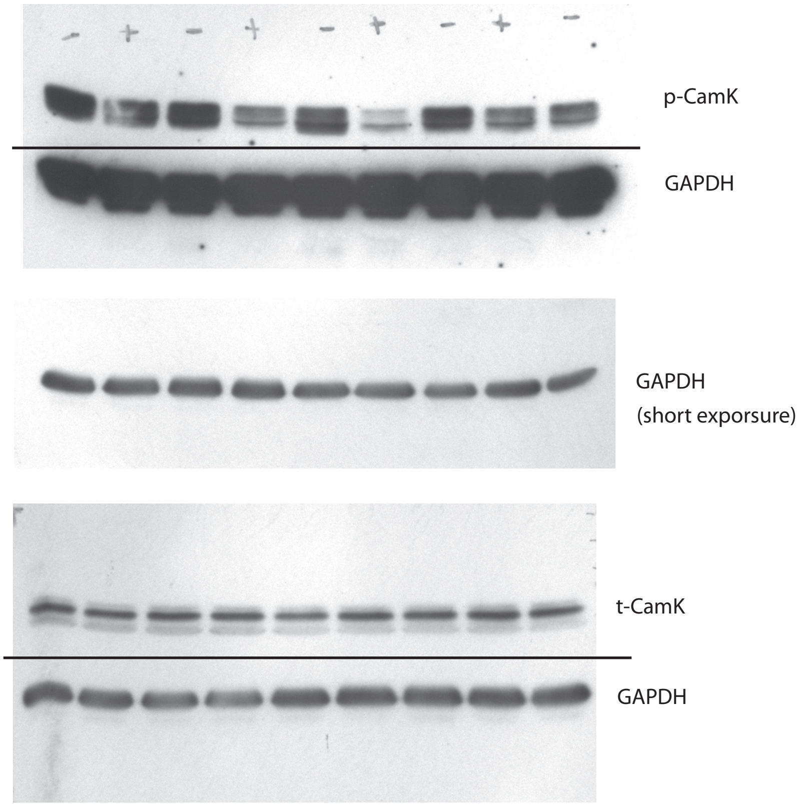

Supplement: Supplementary Information [file srep33853-s1.pdf]
